# The caregiver contribution to self-care of stroke inventory (CC-SCSI): evaluation of psychometric characteristics

**DOI:** 10.1186/s12912-024-01964-3

**Published:** 2024-04-26

**Authors:** Wenna Wang, Zhenxiang Zhang, Yongxia Mei, Bing Zhou, Dudu Zhang, Lamei Liu

**Affiliations:** 1 School of Nursing and Health, Zhengzhou University, Zhengzhou, Henan China; 2 School of Information Engineering, Zhengzhou University, Zhengzhou, Henan China

**Keywords:** Stroke, Caregiver, Contribution, Self-care, Psychometric, Nursing

## Abstract

**Background:**

The caregiver contribution to self-care plays an important role in improving the health outcomes of chronic patients, which needs urgent attention. However, it has been hindered by the lack of a tailored instrument that assesses the caregiver contribution to self-care of stroke.

**Objectives:**

To test the psychometric characteristics of the caregiver contribution to self-care of stroke Inventory (CC-SCSI) in health care practice.

**Methods:**

Participants were recruited in Henan Province from March 2021 to October 2022, utilizing a multicenter stratified sampling approach. A 23-item self-report CC-SCSI with 3 separate scales measuring caregiver contribution to self-care maintenance of stroke, caregiver contribution to self-care monitoring of stroke and caregiver contribution to self-care management of stroke was tested for validity through discriminant validity, confirmatory factor analysis, concurrent validity and convergent validity. The reliability was tested by Cronbach’s α coefficient and test-retest reliability. Qualitative data were analysed using thematic analysis. A STROBE checklist was used.

**Results:**

The three scales of the CC-SCSI exhibited good discriminant validity. Confirmatory factor analysis supported the three-factor structure of the Caregiver Contribution to Stroke Self-Care Maintenance and Management scales and the one-factor structure of the Caregiver Contribution to Stroke Self-Care Monitoring scale. The moderate correlation between the CC-SCSI and the Self-Care of Stroke Inventory indicated acceptable concurrent validity. The moderate correlation between the CC-SCSI and the Caregiver Contribution to Self-Care of Chronic Illness Inventory indicated acceptable convergent validity. Cronbach’s αs for the Caregiver Contribution to Self-Care Maintenance, Self-Care Monitoring, and Self-Care Management scales ranged from 0.876 to 0.974. Test-retest reliability showed average ICC values ranging from 0.828 to 0.903 (*P* < 0.001).

**Conclusions:**

The 23-item CC-SCSI presents good psychometric properties and could be used to explore the caregiver contribution to stroke self-care in health care practice and research.

**Supplementary Information:**

The online version contains supplementary material available at 10.1186/s12912-024-01964-3.

## Introduction

Globally, stroke imposes a significant burden on patients, their families, and society [1]. In 2019, it remained the second-leading cause of death and the third-leading cause of death and disability combined [2]. The incidence rate of stroke varies across countries, ranging from approximately 40 to 250 cases per 100,000 population, and as a result, the number of stroke survivors living with disabilities has surpassed an astonishing 80 million [3]. Promoting self-care has emerged as a key strategy in addressing the burden of stroke [4]. Stroke, being a common chronic illness, often leads to long-term disabilities and various functional impairments in a majority of patients. Self-care in stroke extends beyond individual patient behaviors and involves the collaborative efforts of patients and their caregivers [5]. The caregiver contribution to self-care of stroke exerts a substantial impact on the patient’s self-care process [5, 6]. Therefore, recognizing the crucial role of caregivers in stroke self-care is of utmost importance. However, there is currently a dearth of targeted quantitative instrument for assessing the caregiver contribution to the self-care of stroke.

### Background

Based on the Middle Range Theory of Self-Care of Chronic Illness, self-care in the context of chronic diseases involves the active pursuit of health through health promotion strategies and disease management. It encompasses three essential components: self-care maintenance, self-care monitoring, and self-care management [6, 7]. Systematic review of quantitative studies have demonstrated that self-care could enhance medication adherence among stroke survivors, aid in the recovery of physiological and psychological functions, prevent stroke recurrence, and reduce hospital readmission rates, ultimately improving patients’ quality of life [8]. Furthermore, self-care empowers patients, providing them with a sense of accomplishment and perceived control over the rehabilitation process, leading to increased happiness and well-being [9, 10].

Research has shown that out of the 8760 hours in a year, stroke survivors spend an average of only about 10 hours, or 0.001%, of their time with healthcare professionals [5]. This indicates that the majority of activities related to health maintenance, monitoring, and management are carried out by the patients themselves or in collaboration with their caregivers in an outpatient setting. Self-care in patients with chronic diseases is significantly dependent on the support and assistance provided by caregivers [5, 11]. This relationship is theoretically termed as caregiver contribution to self-care. And it encompasses the process in which caregivers oversee or aid patients in executing self-care actions aimed at sustaining disease stability and symptom management [11]. Caregivers play a crucial role in supporting stroke survivors by providing personal care, emotional support, and practical assistance [12]. They serve as reminders, supervisors, and motivators in the patients’ self-care process. Consequently, the caregiver contribution to self-care of stroke significantly influences their overall health outcomes, underscoring its undeniable importance.

Developing self-care assessment instruments based on the middle range theory of self-care in chronic illness has been highly recommended [13]. Our previous research resulted in the development of the Self-Care of Stroke Inventory, which has demonstrated robust reliability and validity [4]. However, there are currently a limited number of tools available to measure the caregiver contribution to patient self-care. For instance, the Caregiver Contribution to Self-Care of Heart Failure Index (CC-SCHFI) is a 22-item instrument specifically designed to measure the caregiver’s contribution to self-care in heart failure patients [14]. Similarly, the Caregiver Contribution to Self-Care in Ostomy Patient Index (CC-OSCI) is a 22-item instrument developed to measure the caregiver’s contribution to self-care in individuals with colostomy and urostomy [15]. It evaluates the dimensions of self-care maintenance, monitoring, and management in this population. Additionally, the Caregiver Contribution to Self-Care Chronic Illness Inventory (CC-SC-CII) assesses caregiver contribution to self-care in individuals with chronic illnesses such as heart failure, diabetes mellitus, chronic obstructive pulmonary disease (COPD), hypertension, asthma, osteoporosis, and kidney failure [16]. Stroke survivors often face various physical, cognitive, and emotional challenges that distinguish them from patients with other chronic conditions, so the caregiver contribution to self-care of stroke differ from caregiver contribution to self-care of other chronic patients [5, 17]. Based on the existing tools, there is a clear gap in assessing the caregiver contribution to self-care specifically tailored for stroke caregivers.

Caregivers play a significant role in self-care of stroke, but there is currently a lack of suitable instruments to quantify this contribution and understand its relationship with other health-related variables in the stroke population. Accurately measuring the caregiver contribution to self-care of stroke could help explore the specific relationship between patient self-care and caregiver contribution to self-care in the context of dyadic self-care [17]. And it could also clarify impact factors of the caregiver contribution to the self-care of stroke and allow for the examination of the effectiveness of dyadic self-care interventions for stroke patients and caregivers to enhance the refinement of intervention strategies [17, 18]. Consequently, based on our previous tool used to access self-care of stroke survivors [4], it is crucial to develop a corresponding assessment instrument specifically measure the caregiver contribution to self-care of stroke.

## Methods

This study is a psychometric evaluation conducted in 2022. The study report adhered to the Strengthening the Reporting of Observational Studies in Epidemiology (STROBE) statement: guidelines for reporting observational studies [19].

### Aim

The aim of this study was to test and validate the psychometric properties of the caregiver contribution to self-care of stroke Inventory (CC-SCSI).

### Design

A cross-sectional, mixed methods research design was adopted, involving the collection of quantitative and qualitative data. Participants were recruited in Henan Province from March 2021 to October 2022 utilizing a multicenter stratified sampling approach to collect quantitative data. Five cities were randomly selected from each of the Eastern, Western, Southern, Northern, and Central regions of Henan Province in China. Within the five selected cities, five administrative districts were randomly chosen. From these five administrative districts, research subjects who met the inclusion criteria were selected for the questionnaire survey. Quantitative data enabled calculation of construct validity, discriminant validity, concurrent validity, convergent validity and reliability of the instrument. The qualitative data presented reflections from respondents, offering feedback regarding the challenges they encountered while using it and providing valuable suggestions for improvement.

The Self-Care of Stroke Inventory (SCSI) is a measure of self-care in stroke survivors developed in our previous study [4](Supplementary file 1). It includes 23 items divided into three separate scales: self-care maintenance (9 items), self-care monitoring (6 items), and self-care management (8 items) scales. In the context of this study, the Caregiver Contribution to Self-Care of Stroke Inventory (CC-SCSI) designed in Simplified Chinese was based on the Self-Care of Stroke Inventory (SCSI) to make it applicable for measurement within the caregiver population (Supplementary file 2). The CC-SCSI is also a 23-item instrument that measures the caregiver contribution to self-care of stroke. Each item uses a 5-point Likert scale for responses from “Never” to “Always”. Higher scores indicate a higher level of self-care contribution. The CC-SCSI aligns with the SCSI in item number and content but differs in questioning format, providing an accurate and comprehensive measure of the caregiver contribution to self-care of stroke.

### Participants and setting

The potential participants were identified and recruited from the community with the guidance of the staff from community health service centers in 5 cities across Henan Province. Eligibility criteria for stroke caregivers in this study included the following: (1) the patients they care for are individuals who have been definitively diagnosed with stroke, as documented in medical records; (2) The patients being cared for have stable medical conditions and no significant cognitive impairments (as assessed by the Mini-Mental State Examination, with an MMSE score of ≥21 points); (3) Informal caregivers (such as spouses, children, parents, etc., of the patients) who have been continuously caring for stroke survivors for more than 1 month; (4) aged 18 or above; (5) Demonstrating adequate cognitive and communication abilities to provide informed consent and participate in the survey.

### Data collection

A questionnaire was used to obtained general information, including age, gender, relationship with the patient, marital status, educational level, employment status, daily caregiving duration, total caregiving duration, presence of any chronic illnesses, etc. The surveyors in this study were trained individuals who were not directly involved in the research process. There was no personal or vested interest between the surveyors and the participants, ensuring the objectivity and integrity of the research. They clearly communicated the purpose and significance of the study to the participants, ensuring their voluntary participation and guaranteeing anonymity and confidentiality. Surveyors diligently reviewed the questionnaires for completeness and promptly addressed any concerns or provided clarifications either during face-to-face interactions or through phone conversations. In addition, for the qualitative research aspect, a semi-structured interview method was utilized. These interviews were conducted after the completion of the questionnaire survey, and the point of data saturation determined when the interviews were concluded.

### Data analysis

Questionnaires were consolidated into a single database using IBM SPSS Statistics version 25. Confirmatory factor analysis (CFA) was conducted using AMOS 24.0.

#### Quantitative analyses

##### Descriptive statistics

In this study, univariate (standardized score) and multivariate outliers in the dataset were examined. Following the common recommendation, these outliers were excluded from all subsequent analyses due to their potential influence on the confirmatory factor analysis (CFA). Descriptive statistics, including mean, standard deviation (SD), and frequency, were calculated for the variables of interest. Skewness and kurtosis of the CC- SCSI items were assessed to determine the most appropriate CFA estimator for the second phase of analysis [16].

##### Discriminant validity

To assess the discriminant validity of the CC-SCSI and determine its ability to detect differences between different caregiver characteristics (such as current employment status, relationship with the patient and daily care duration) were considered. Subgroups based on caregiver characteristics were subjected to one-way ANOVA (for differences between more than two groups) or independent samples t-tests (for differences between two groups) to analyze the scores.

##### Construct validity

A confirmatory factor analysis (CFA) was conducted to assess the goodness-of-fit of the factor structure. The overall fit of the model was evaluated using several indices, including the chi-square goodness-of-fit test, chi-square to degrees of freedom ratio (χ^2^/df), goodness-of-fit index (GFI), adjusted goodness-of-fit index (AGFI), comparative fit index (CFI), Tucker-Lewis index (TLI), incremental fit index (IFI), root mean square error of approximation (RMSEA), and root mean square residual (RMR).

The χ^2^/df value below 5 indicates an acceptable fit for the model. The values of the GFI and AGFI were > 0.80, indicating that the model was acceptable. Additionally, the RMSEA value being less than 0.08 and the RMR being less than 0.05 also contribute to the model’s acceptability. Regarding the CFI, TLI, and IFI, values exceeding 0.90 indicate a good fit between the model path and the observed data [20, 21].

##### Concurrent validity, convergent validity

The Self-Care of Stroke Inventory (SCSI) [4] developed in our previous study was used as a comparison instrument to assess the convergent validity of the CC-SCSI scale. By comparing the CC-SCSI with the SCSI, the consistency between the two instruments in measuring the same or similar concepts can be determined. Additionally, the Caregiver Contribution to Self-Care of Chronic Illness Inventory (CC-SC-CII) developed by Vellone et al. was used as a comparison instrument to assess the concurrent validity of the CC-SCSI scale [16]. By comparing the CC-SCSI with the CC-SC-CII, the correlation between the two instruments can be evaluated, providing insights into the consistency of assessing caregiver contributions to self-care.

##### Reliability

Cronbach’s alpha coefficient (Cronbach’s α) was calculated for three scales and each of their dimensions. A Cronbach’s α value above 0.7 was considered indicative of good reliability, as suggested by Tavakol et al. [22]. Test-retest reliability was assessed using the Intraclass Correlation Coefficient (ICC) with a 95% confidence interval (CI) [23]. Reproducibility was categorized as “excellent” if the ICC was greater than 0.75, “good” if the ICC ranged from 0.60 to 0.74, “fair” if the ICC ranged from 0.40 to 0.59, and “poor” if the ICC was less than 0.40 [24]. And the sample size for retesting is generally no less than 10% of the total sample.

#### Qualitative analysis

Thematic analysis [25] was employed for analyzing qualitative responses to the questions (Supplementary file 3) like (1) What are your thoughts on this instrument? Was it helpful? (2) Could you explain the specific challenges you faced in understanding certain parts of the questionnaire?. Then the researchers analyzed the data by open coding and searched for themes.

### Ethical considerations

This study was approved by the Research Ethics Committee of Zhengzhou University (ZZUIRB2021–115). Participation was voluntary, and no identifiable data were gathered, so anonymity and confidentiality were assured. All participants provided their consent to participate either in written or verbal form.

## Results

### Respondents

Among the 574 eligible caregivers identified by the researchers, 530 (92.5%) agreed to participate and successfully completed the questionnaires, while 44 (7.5%) declined to participate due to reasons such as lack of time or interest. A total of 60 out of the 530 stroke caregivers indicated their interest and participated in the retest to assess the test-retest reliability of the scale. Table 1 presents the characteristics of the participants (*n* = 530). A significant majority of stroke caregivers were female (74.0%), married (95.3%), and either resigned, unemployed, or retired (75.4%). Additionally, 72.1% had a spousal relationship with the patient, 66.8% reported a daily care duration exceeding 8 hours, and 64.3% did not have any chronic diseases. More than half of the stroke caregivers (63.2%) had an educational background of junior high school or below, with 39.4% having a junior high school education and 23.8% having primary school education or below. Table 1 presents the demographic characteristics of the participants. In addition, on average, stroke survivors were more than 10 years older than their caregivers and most were male (72.5%).

**Table 1:** Caregivers’ characteristics and patients’ characteristics (*n* = 530)

Characteristics	M ± SD/ n (%)	Characteristics	M ± SD/ n (%)
	**Caregivers**		**Stroke survivors**
Age	57.02 ± 12.859	Age	62.74 ± 11.674
Gender		Gender	
Male	138(26.0)	Male	384(72.5)
Female	392(74.0)	Female	146(27.5)
Educational Level		Educational Level	
Primary school or below	126(23.8)	Primary school or below	150(28.3)
Junior high school	209(39.4)	Junior high school	178(33.6)
High school	130(24.5)	High school	119(22.4)
College or above	65(12.3)	College or above	83(15.7)
Marital Status		Marital Status	
Unmarried	22(4.2)	Unmarried	15(2.8)
Married	505(95.3)	Married	464(87.6)
Divorced/Widowed	3(0.6)	Divorced/Widowed	51(9.6)
Current Employment Status		Disease Duration	
Employed	130(24.5)	< 3 months	131(24.7)
Resigned/ Unemployed	200(37.7)	3 months ~ 1 year	85(16.0)
Retired	200(37.7)	1 year ~ 3 years	109(20.6)
Average Monthly Family Income		3 years and above	205(38.7)
Less than needed	214(40.4)	Stroke Type	
Enough for living	205(38.7)	Ischemic	415(78.3)
More than needed	111(20.9)	Hemorrhagic	81(15.3)
Relationship with the Patient		Mixed type	34(6.4)
Spouse	382(72.1)	Number of Strokes	
Children	120(22.6)	1	247(46.6)
Parents	16(3.0)	2	162(30.6)
Relatives/Friends	12(2.3)	3~	121(22.8)
Daily Care Duration		Modified Barthel Index (MBI)	74.22 ± 20.624
Less than 4 hours	57(10.8)		
4 to 8 hours	119(22.4)		
More than 8 hours	354(66.8)		
Total Care Duration			
Less than 3 months	136(25.7)		
3 months to 1 year	82(15.5)		
1 year to 3 years	103(19.4)		
3 years and above	209(39.4)		
Presence of Chronic Diseases	79(25.8)		
Yes	189(35.7)		
No	341(64.3)		

*M* mean, *SD* Standard Deviation

### Validity of the instrument

#### Descriptive analyses of items

Table 2 presents the mean, standard deviation (SD), skewness, and kurtosis values for the CC-SCSI items. The skewness values of the items vary from approximately − 0.997 to 0.364, while the kurtosis values range from approximately − 1.373 to 0.382. Among the Caregiver Contribution to Self-Care Maintenance, Monitoring, and Management scales, the items with the highest scores were “Recommend/help the patient to take medicines following the doctor’s advice,” “Recommend/help the patient to monitor the signs and symptoms of stroke occurrence/recurrence,” and “Recommend/help the patient to do daily activities (e.g., washing, housework) within one’s ability, as permitted by physical condition,” respectively. On the other hand, the items with the lowest scores were “Recommend/help the patient to obtain stroke-related information through multiple channels,” “Recommend/help the patient to monitor the occurrence and development of stroke sequelae/complications,” and “Recommend/help the patient to do something to relieve stress and negative emotions.”

**Table 2:** Descriptive statistics of the CC-SCSI items

Item	M	SD	Skewness	Kurtosis	Factor loading
Caregiver Contribution to Self-Care Maintenance scale					
The following are behaviors that stroke survivors may adopt to promote physical and mental health. Please indicate how often do you recommend the patient you care to do the following behaviors? Or how often do you help the patient you care when he/she cannot do it independently?					
1. Recommend /help the patient to maintain normal weight (height/weight 2 = 18.5 ~ 24 kg/m^2^)	3.52	1.367	−0.665	− 0.783	0.615
2. Recommend /help the patient to maintain over 30 minutes mild or moderate physical activity 3 or more times a week	3.72	1.145	−0.78	− 0.057	0.808
3. Recommend /help the patient to maintain healthy eating habits (e.g., Salt intake < 6 g/d, have more fruits and vegetables)	3.84	1.083	−0.942	0.382	0.818
4. Recommend /help the patient to maintain a regular life schedule (e.g., early to bed and early to rise, avoid excessive fatigue)	3.77	1.122	−0.859	0.096	0.742
5. Recommend /help the patient to master stroke-related information (e.g., etiology, outcome, treatment and rehabilitation of stroke)	2.77	1.439	0.137	−1.373	0.924
6. Recommend /help the patient to obtain stroke-related information through multiple channels (e.g., mobile terminals, books, consultants)	2.55	1.319	0.364	−1.064	0.926
7. Recommend /help the patient to take medicines following the doctor advised	3.87	1.137	−0.918	0.073	0.839
8. Recommend /help the patient to do rehabilitation exercises following the doctor advised	3.86	1.145	−0.903	0.031	0.837
9. Recommend /help the patient to do regular check-ups following the doctor advised	3.84	1.195	−0.997	0.157	0.881
**Caregiver Contribution to Self-Care Monitoring scale**					
The following are behaviors that stroke survivors may adopt to monitor their condition. Please indicate how often do you recommend the patient you care to do the following behaviors? Or how often do you help the patient you care when he/she cannot do it independently?					
10. Recommend /help the patient to monitor the signs and symptoms of stroke occurrence / recurrence (e.g., dizzy, eye and mouth distortion, limb weakness on one side)	3.43	1.413	− 0.493	−1.094	0.858
11. Recommend /help the patient to monitor the occurrence and development of stroke sequelae/complications (e.g., hemiplegia, pneumonia, pressure sores)	3.23	1.429	−0.223	−1.311	0.879
12. Recommend /help the patient to monitor symptoms during daily activities (e.g., fatigue, pain)	3.38	1.387	−0.379	−1.148	0.920
13. Recommend /help the patient to monitor changes in biochemical indicators such as blood pressure and blood sugar	3.29	1.338	−0.272	−1.127	0.879
14. Recommend /help the patient to monitor medication effects and side effects	3.30	1.342	−0.284	−1.122	0.905
15. Recommend /help the patient to monitor the effect of rehabilitation exercise	3.31	1.362	−0.321	−1.122	0.880
**Caregiver Contribution to Self-Care Management scale**					
The following are behaviors that stroke survivors may adopt to control physical symptoms and emotional changes. Please indicate how often you would recommend/help the patient you care for to do the following behaviors when they cannot do it independently when they experience symptoms or emotional changes?					
16. Help the patient to correctly respond to emergency situations such as stroke occurrence / recurrence	3.53	1.273	−0.752	−0.498	0.938
17. Help the patient to correctly handle stroke sequelae / complications	3.48	1.263	−0.683	−0.518	0.952
18. Help the patient to inform the medical staff as soon as possible when medication side effects occur	3.82	1.058	−0.876	0.300	0.750
19. Recommend /help the patient to do daily activities (e.g., washing, housework) within one’s ability, as permitted by physical condition	3.91	1.038	−0.847	0.177	0.875
20. Recommend /help the patient to participate in social life (e.g., participation in parties, return to work), as permitted by physical condition	3.75	1.110	−0.691	−0.254	0.894
21. Recommend /help the patient to adjust the way and intensity of exercise flexibly according to the condition, rehabilitation effect and environmental conditions	3.74	1.157	−0.715	−0.262	0.872
22. Recommend /help the patient to develop a positive attitude towards life (e.g., avoid the idea that you are a burden, keep smiling)	3.64	1.148	−0.633	−0.309	0.924
23. Recommend /help the patient to do something to relieve stress and negative emotions (e.g., find someone to talk to, listen to music)	3.42	1.282	−0.458	−0.761	0.931

*M* mean, *SD* Standard Deviation

#### Discriminant validity

In the Caregiver Contribution to Self-Care Maintenance scale, there is a significant difference in mean scores between gender (t = 10.439, *P* = 0.001), educational level (F = 3.379, *P* = 0.018), current employment status (F = 3.995, *P* = 0.037), relationship with the patient (F = 5.132, *P* = 0.002), and daily care duration of the caregivers (F = 10.681, *P* < 0.001). In the Caregiver Contribution to Self-Care Monitoring scale, there is a significant difference in mean scores between gender (t = 8.551, *P* = 0.004), educational level (F = 4.229, *P* = 0.006), current employment status (F = 8.498, *P* < 0.001), relationship with the patient (F = 2.917, *P* = 0.046), and daily care duration of the caregivers (F = 11.819, P < 0.001). In the Caregiver Contribution to Self-Care Management scale, there is a significant difference in mean scores between educational level (F = 7.196, P < 0.001), current employment status (F = 7.405, *P* = 0.001), relationship with the patient (F = 3.348 *P* = 0.019), and daily care duration of the caregivers (F = 6.116, P < 0.001). This indicates that the three scales exhibit good discriminant validity.

#### Construct validity

To ensure consistency with previous validation studies conducted using other instruments inspired by the Middle Range Theory of Self-Care of Chronic Illness (Vellone, Lorini, et al., 2020; W. Wang et al., 2023), the researchers performed three separate Confirmatory Factor Analyses (CFAs), one for each scale. During the CFA, the same factorial structure that was previously validated for the SCSI was tested (i.e., the same items were grouped in the same factors and scales). This decision was made because the CC-SCSI shares the same theoretical underpinning as the SCSI.

##### Caregiver contribution to self-care maintenance scale

**Fig. 1:**
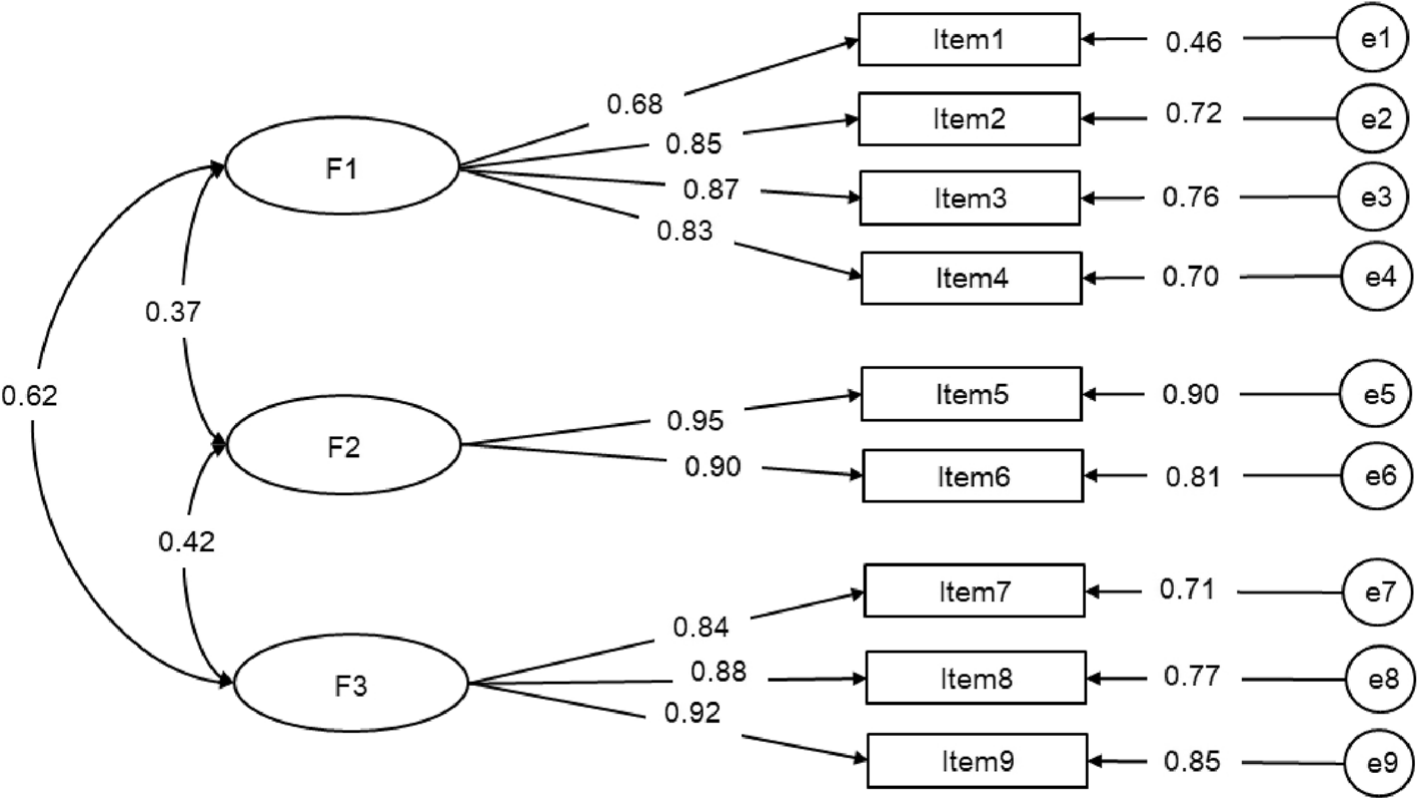
Measurement of the three-factor model of the Caregiver Contribution to Self-Care Maintenance scale via CFA (n = 530). Note. F1 (factor 1): caregiver contribution to daily health behaviors, F2 (factor 2): caregiver contribution to knowledge gaining, F3 (factor 3): caregiver contribution to treatment compliance

The researchers specified a three-factor model for the Caregiver Contribution to Self-Care Maintenance scale. The three factors were identified as follows: caregiver contribution to daily health behaviors (items 1 to 4), caregiver contribution to knowledge gaining (items 5 and 6), and caregiver contribution to treatment compliance (items 7 to 9). The results of the confirmatory factor analysis of the scale, which comprised 9 items, are shown in Fig. 1. All factor loadings were significant (*P* < 0.05) and generally medium to high (ranging from 0.68–0.95). The final results of the three-factor model were as follows: χ^2^/df = 2.771, RMSEA = 0.058, GFI = 0.976, AGFI = 0.951, RMR = 0.041, CFI = 0.989, TLI = 0.981, IFI = 0.989.

##### Caregiver contribution to self-care monitoring scale

**Fig. 2:**
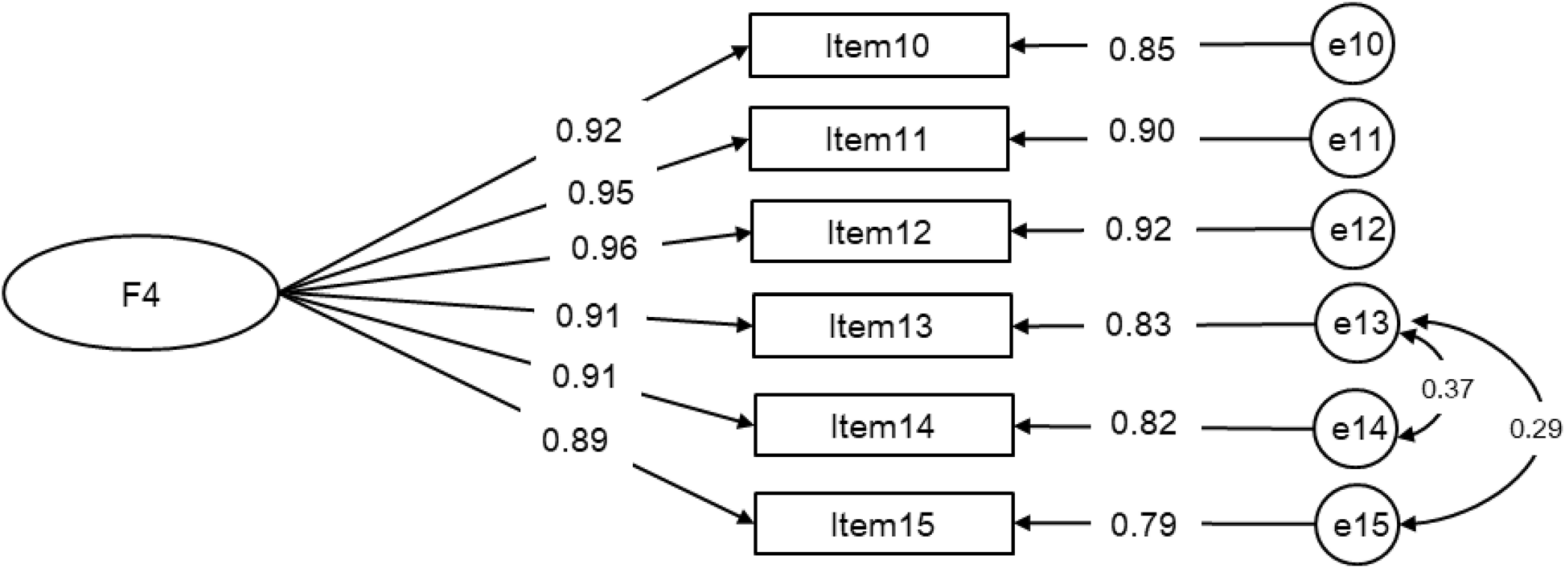
Measurement of the one-factor model of the Caregiver Contribution to Self-Care Monitoring scale via CFA (*n* = 530). Note. F4 (factor 4): caregiver contribution to self-care monitoring

In the study, the researchers specified a unidimensional factor model for the Caregiver Contribution to Self-Care Monitoring Scale, which included items 10 through 15, yielded a poor fit: χ^2^/df = 9.050, RMSEA = 0.123, GFI = 0.960, AGFI = 0.879, RMR = 0.027, CFI = 0.988, TLI = 0.9974, IFI = 0.988. After Controlling the correlations of residuals between items 13 and 14, items 13 and 15, the model fit was observed to be favorable, with the following indices: χ^2^/df = 3.440, RMSEA = 0.068, GFI = 0.989, AGFI = 0.953, RMR = 0.013, CFI = 0.997, TLI = 0.992, IFI = 0.997. All factor loadings were significant (*P* < 0.05) and generally high (ranging from 0.89–0.96).Detailed information and results can be found in Fig. 2**.**

##### Caregiver contribution to self-care management scale

**Fig. 3:**
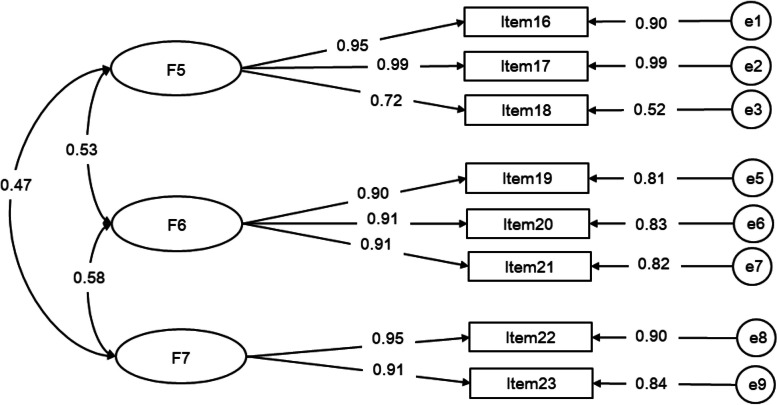
Measurement of the three-factor model of the Caregiver Contribution to Self-Care Management scale via CFA (n = 530). Note. F5 (factor 5): caregiver contribution to symptom management, F6 (factor 6): caregiver contribution to activities and rehabilitation management, F7 (factor 7): caregiver contribution to emotional management

Regarding the Caregiver Contribution to Self-Care Management scale, we defined three factors: “caregiver contribution to symptom management” (items 16 to 18), “caregiver contribution to activities and rehabilitation management” (items 19 to 21), and “caregiver contribution to emotional management” (items 22 and 23). All standardized estimates/regression weights (the numbers in the figure) are above 0.7, indicating strong relationships between the latent variables and their respective indicators. Furthermore, the fit indices demonstrated a favorable fit for the three-factor structure: χ^2^/df = 3.601, RMSEA = 0.070, GFI = 0.980, AGFI = 0.941, RMR = 0.03, CFI = 0.993, TLI = 0.983, IFI = 0.993. For more detailed information, please refer to Fig. 3.

#### Concurrent validity, convergent validity

The correlation analysis revealed a moderate positive association between the three scales of CC-SCSI and the corresponding three scales of SCSI, with r values of 0.317, 0.548, and 0.324 (All *P* < 0.001). Similarly, the correlation between the three scales of CC-SCSI and those of CC-SC-CII was also moderately positively correlated, with r values of 0.412, 0.568, and 0.394 (All *P* < 0.001).

### Reliability

In the Caregiver Contribution to Self-Care Maintenance scale, the Cronbach’s α of the scale was 0.884, with each dimension of the scale having Cronbach’s αs ranging from 0.876 to 0.918. The test-retest reliability showed an average measure of ICC of the Caregiver Contribution to Self-Care Maintenance scale as 0.903 (95% CI [0.798–0.954], *P* < 0.001). For the Caregiver Contribution to Self-care Monitoring scale, the overall Cronbach’s α was 0.974. The test-retest reliability had an average measure of ICC of the Caregiver Contribution to Self-care Monitoring scale at 0.855 (95% CI [0.698–0.931], P < 0.001). Regarding the Caregiver Contribution to Self-care Management scale, the Cronbach’s α of the scale was 0.910, and each dimension of the scale had Cronbach’s αs ranging from 0.916 to 0.931. The test-retest reliability yielded an average measure of ICC of the Caregiver Contribution to Self-care Management scale as 0.828 (95% CI [0.641–0.918], P < 0.001). The results of this analysis are presented in Table 3.

**Table 3:** Reliability of the CC-SCSI (n = 530)

The Caregiver Contribution to Self-Care of Stroke Inventory (CC-SCSI)	Number of items	Cronbach’s α	test-retest reliability (ICC, 95%CI)
**Caregiver Contribution to Self-care Maintenance scale**	9	0.884	0.903,[0.798–0.954]
Caregiver Contribution to daily health behaviors	4	0.876	0.840,[0.667–0.924]
Caregiver Contribution to knowledge gaining	2	0.918	0.901,[0.794–0.953]
Caregiver Contribution to treatment compliance	3	0.910	0.879,[0.748–0.942]
**Caregiver Contribution to Self-care Monitoring scale**	6	0.974	0.855,[0.698–0.931]
**Caregiver Contribution to Self-care Management scale**	8	0.910	0.937,[0.870–0.970]
Caregiver Contribution to symptom management	3	0.917	0.875,[0.739–0.94]
Caregiver Contribution to activities and rehabilitation management	3	0.931	0.969,[0.936–0.985]
Caregiver Contribution to emotional management	2	0.916	0.828,[0.641–0.918]

### Caregiver reflections on the instrument

This study conducted interviews with 15 caregivers and obtained reflections and experiences on the use of the instrument through an interview guide including several open-ended questions (Supplementary file 3). Through thematic analysis, three main points were distilled, as shown in Table 4. Based on caregivers’ feedback, the following modifications were made to the questionnaire. Provide explanations for some items that were difficult to understand, such as revising item 2 to “ Item 2 Recommend /help the patient to maintain over 30 minutes mild or moderate physical activity 3 or more times a week ( e.g. continuous walking, stair climbing) “. Include reminders when distributing the questionnaire to avoid misconceptions, for example, for item 7, emphasize that “take medicines following the doctor advised” not only refers to taking medication on time but also involves aspects like avoiding missed doses or self-adjusting medication dosage. Consider using Mandarin, dialects, or plain language depending on the participants’ preferences when administering the questionnaire.

**Table 4:** Caregiver reflections on the instrument

Benefits and Usefulness	Difficulties and Challenges	Suggestions for improvement
The instrument proved helpful in enabling caregivers to identify the specific aspects of self-care support where they may be inadequate in providing for the patients, with a particular focus on self-care monitoring.	Some items in the questionnaire were difficult to understand, such as item 2, which asked about “moderate physical activity.” Participants wished for clearer definitions or examples for such terms.	Provide clearer definitions or examples for terms and concepts in the questionnaire to enhance understanding.
Filling out this instrument has given me time to think about my contribution to patients and relieved me of anxiety and guilt.	The questionnaire was perceived as lengthy, and some caregivers lacked the patience to answer all the questions at once.	When distributing the questionnaire, try to avoid the busy time of the caregiver, and distribute the questionnaire when the caregiver is free and energetic.
This instrument is for caregivers and it made me see my worth to stroke survivors.	Participants found it challenging to judge the frequency of certain behaviors, as it felt too subjective and uncertain if they were filling it out correctly.	Improve the objectivity of frequency assessment to make it easier for caregivers to respond accurately.

### Final instrument

The Caregiver Contribution to Self-Care of Stroke Inventory (CC-SCSI) is comprised of 23 items, organized into 7 dimensions and 3 independent scales. All items are rated on a 5-point Likert scale, where 1 represents “never” and 5 represents “always.” Each CC-SCSI scale is assigned a standardized score ranging from 0 to 100. The Caregiver Contribution to Self-Care Maintenance scale consists of 3 dimensions with a total of 9 items, and the total score = (sum of items 1 to 9–9) × (100/36); The Caregiver Contribution to Self-Care Monitoring scale contains 1 dimension comprising 6 items, and the total score = (sum of items 1 to 6–6) × (100/24); The Caregiver Contribution to Self-Care Management scale is made up of 3 dimensions with a total of 8 items, and the total score = (sum of items 1 to 8–8) × (100/32). The CC-SCSI could be used to assess the current level of caregiver contribution to self-care of stroke survivors. A higher scale score indicates a greater level of caregiver contribution to the self-care of stroke survivors.

## Discussion

Due to the critical importance of stroke patients’ self-care for their health outcomes and the challenges they face in performing self-care due to residual functional impairments, measuring the caregiver contribution to self-care of stroke is highly meaningful [5]. The objective of this study was to examine the psychometric properties of the Caregiver Contribution to Stroke Self-Care Inventory. This was accomplished by conducting statistical and qualitative analyses of the feedback provided by caregivers. The use of a mixed research method enhanced the scientific rigor, reliability, and effectiveness of the process in developing this scale [26].

The psychometric evaluation of the Caregiver Contribution to Self-Care of Stroke Inventory (CC-SCSI) provided valuable insights into the instrument’s validity and reliability. The high response rate (92.5%) indicates strong engagement and interest among stroke caregivers in participating in this study. The majority of caregivers were female, reflecting the common gender distribution of caregivers in stroke care. Moreover, the caregivers were found to be closely related to the stroke survivors, with spousal relationships being predominant, highlighting the crucial role of family caregivers in the stroke care context.

The descriptive analysis of the CC-SCSI items revealed varying levels of caregiver contributions to different aspects of stroke self-care. Caregivers demonstrated active contribution in promoting medication adherence, monitoring stroke-related signs and symptoms, and assisting with daily activities. However, they reported lower levels of contribution to activities such as seeking stroke-related information, monitoring sequelae/complications, and helping stroke survivors manage emotional well-being. These findings underscore the need to focus on enhancing caregiver support in these specific areas to improve stroke survivors’ self-care.

The discriminant validity analysis demonstrated that the CC-SCSI effectively differentiated caregiver contributions based on gender, educational level, employment status, relationship with the patient, and caregiving duration. This suggests that the instrument can accurately measure distinct aspects of caregiver involvement in stroke self-care, making it a useful tool for tailoring interventions to individual caregiver needs. Based on the discriminant validity results, healthcare providers can design tailored education programs for different caregiver subgroups, taking into account factors such as gender, educational level, and daily caregiving duration. Personalized educational initiatives can empower caregivers with the necessary skills and knowledge to effectively support stroke survivors’ self-care [27].

The construct validity analysis using Confirmatory Factor Analysis (CFA) provided evidence supporting the three-factor structure of the CC-SCSI, which aligns with the Middle Range Theory of Self-Care of Chronic Illness. This indicates that the instrument measures the intended constructs of caregiver contributions to self-care maintenance, monitoring, and management. The favorable fit indices confirm the validity of the scale, further validating its applicability in the stroke care context. In addition, the CFA results of this study demonstrated a good fit for the CC-SCSI data, similar to the SCSI [4]. These findings further support the notion that the involvement of caregivers in the self-care process for chronic patients is comparable [28].

The findings reveal that the CC-SCSI exhibits good convergent validity when compared to the SCSI [4], as evidenced by moderate positive associations between their corresponding scales (r values of 0.317, 0.548, and 0.324; all *P* < 0.001). Furthermore, the CC-SCSI’s concurrent validity was assessed against the CC-SC-CII [16], revealing similarly moderate positive correlations (r values of 0.412, 0.568, and 0.394; all P < 0.001). Overall, the robust correlations observed in both convergent and concurrent validity assessments support the validity of the CC-SCSI as a comprehensive tool for evaluating the caregiver contribution to self-care in stroke context.

Reliability refers to the consistency, stability, and dependability of a measurement or test in producing consistent results when administered to the same individuals under similar conditions [23]. The reliability assessments in this study demonstrated high internal consistency and stability for the three scales of the CC-SCSI. Cronbach’s alpha values were 0.884, 0.974, and 0.910, respectively, indicating strong internal reliability for each scale. Additionally, the test-retest reliability scores were 0.903, 0.855, and 0.828, respectively, suggesting consistent and stable measurements over time. In comparison, the CC-SC-CII developed in Italy [16] reported Cronbach’s α of 0.83, 0.92, and 0.76 for its three scales, exhibiting slightly lower Cronbach’s α values across its scales compared to the CC-SCSI. These findings reinforce the robustness of the CC-SCSI instrument, ensuring its consistency in measuring caregiver contributions to stroke self-care.

Overall, the CC-SCSI offers a reliable and comprehensive assessment tool for evaluating the caregiver contribution to self-care of stroke. The psychometric evaluation of the CC-SCSI in this study highlights the critical role of caregivers in supporting self-care of stroke survivors. The robust psychometric properties and practical applicability of the CC-SCSI position it as a valuable tool for both healthcare providers and researchers. This instrument not only facilitates advancements in the measurement and its key influential factors of caregiver contribution to self-care stroke but also supports the design of tailored dyadic self-care interventions aimed at enhancing health outcomes for both stroke patients and their caregivers [29, 30].

### Limitations

The study has several limitations. Firstly, the research did not employ random sampling to collect samples and test the reliability and validity of the Stroke Caregiver Self-Care Contribution Scale, which may have impacted the reliability of the assessment tool to some extent. However, the study attempted to select stroke caregivers from diverse regions, which improves the generalizability of the research findings to some degree. Secondly, the qualitative research component only served as a supplementary part to the quantitative findings, without deeply integrating and discussing the two aspects. Lastly, the included caregivers in the study provided care for stroke patients with varying durations of illness, and future research could examine the tool’s reliability separately at different stages of the illness trajectory.

## Conclusions

In conclusion, the psychometric evaluation of the Caregiver Contribution to Self-Care of Stroke Inventory (CC-SCSI) demonstrates its validity and reliability as a comprehensive tool to measure the caregiver contribution to self-care of stroke. The instrument effectively captures diverse aspects of caregiver contribution to self-care of stroke, including self-care maintenance, self-care monitoring, and self-care management. Moreover, this instrument could provide an evaluative means for future research exploring dyadic self-care interactions or testing the effectiveness of dyadic self-care intervention programs.

### Relevance to clinical practice

The findings of the study have substantial implications for clinical practices in stroke care. Healthcare professionals should acknowledge the crucial role of caregivers in supporting stroke survivors’ self-care and develop targeted interventions to address specific areas where caregivers may need additional support. Providing caregiver education and training on accessing relevant stroke-related information, monitoring complications, and promoting emotional well-being may help to enhance stroke self-care. Integrating the CC-SCSI into routine clinical assessments enables healthcare professionals to measure the caregiver contribution to stroke self-care regularly. Monitoring the caregiver contribution over time facilitates timely intervention and additional support as needed. Encouraging open communication and collaboration between caregivers and stroke survivors fosters a more supportive and effective self-care partnership. Moreover, recognizing the interconnectedness between stroke survivors and caregivers highlights the importance of adopting a dyadic approach to self-care in interventions.

### Supplementary Information

**Supplementary Material 1.**

**Supplementary Material 2.**

**Supplementary Material 3.**

**Supplementary Material 4.**

## Data Availability

The datasets used in this study are available from the corresponding author upon request.
