# Supplementary material for: The caregiver contribution to self-care of stroke inventory (CC-SCSI): evaluation of psychometric characteristics
Source: BMC Nurs. 2024 Apr 26;23:284. doi: 10.1186/s12912-024-01964-3 (PMC11055333; doi:10.1186/s12912-024-01964-3)
Supplement: Supplementary file 2 — Supplementary Material 2. [file 12912_2024_1964_MOESM2_ESM.docx]

**Supplementary file 1**

The Self-Care of Stroke Inventory (SCSI) was developed in our previous research and has been validated, demonstrating robust reliability and validity ^[1]^.

[1] Wang W, Mei Y, Vellone E, Zhang Z, Liu B, Zhou C, et al. Development and psychometric testing of the self-care of stroke inventory. Disabil Rehabil. 2023:1-10

**The Self-Care of Stroke Inventory (SCSI)**

Instructions：Please think about your experiences last month while completing this survey.

**Part A (Self-Care Maintenance):** The following behaviors may be adopted by stroke survivors to promote physical and mental health. Please indicate how often you engage in the following behaviors by placing a check mark (√) on the corresponding number.

|  | Items | Never |  |  |  | Always |
| --- | --- | --- | --- | --- | --- | --- |
| A-1 Daily health behaviors | 1. Maintain normal weight (height/weight^2^= 18.5~24 kg/m²) | 1 | 2 | 3 | 4 | 5 |
|  | 2. Maintain over 30 minutes mild or moderate physical activity 3 or more times a week ( e.g. continuous walking, stair climbing ) | 1 | 2 | 3 | 4 | 5 |
|  | 3. Maintain healthy eating habits (e.g., Salt intake < 6g/d, have more fruits and vegetables) | 1 | 2 | 3 | 4 | 5 |
|  | 4. Maintain a regular life schedule (e.g., early to bed and early to rise, avoid excessive fatigue) | 1 | 2 | 3 | 4 | 5 |
| A-2 Knowledge gaining | 5. Master stroke-related information (e.g., etiology, outcome, treatment and rehabilitation of stroke) | 1 | 2 | 3 | 4 | 5 |
|  | 6. Obtain stroke-related information through multiple channels ( e.g., mobile terminals, books, consultants ) | 1 | 2 | 3 | 4 | 5 |
| A-3 Treatment compliance | 7. Take medicines following the doctor advised | 1 | 2 | 3 | 4 | 5 |
|  | 8. Do rehabilitation exercises following the doctor advised | 1 | 2 | 3 | 4 | 5 |
|  | 9. Do regular check-ups following the doctor advised | 1 | 2 | 3 | 4 | 5 |

**Part B (Self-Care Monitor):** The following behaviors are adopted by stroke survivors to monitor their condition. Please indicate how often you engage in the following behaviors by placing a check mark (√) on the corresponding number.

|  | Items | Never |  |  |  | Always |
| --- | --- | --- | --- | --- | --- | --- |
| B-1 Self-care Monitor | 10. Monitor the signs and symptoms of stroke occurrence / recurrence ( e.g., dizzy, eye and mouth distortion, limb weakness on one side ) | 1 | 2 | 3 | 4 | 5 |
|  | 11. Monitor the occurrence and development of stroke sequelae/complications(e.g., hemiplegia, pneumonia, pressure sores) | 1 | 2 | 3 | 4 | 5 |
|  | 12. Monitor symptoms during daily activities (e.g., fatigue, pain) | 1 | 2 | 3 | 4 | 5 |
|  | 13. Monitor changes in biochemical indicators such as blood pressure and blood sugar | 1 | 2 | 3 | 4 | 5 |
|  | 14. Monitor medication effects and side effects | 1 | 2 | 3 | 4 | 5 |
|  | 15.Monitor the effect of rehabilitation exercise | 1 | 2 | 3 | 4 | 5 |

**Part C (Self-Care Management):** The following behaviors are adopted by stroke survivors to control physical symptoms and emotional changes. When you experience symptoms or emotional changes, how likely are you to take the following measures? For each measure, please place a check mark (√) on the corresponding number.

|  | Items | Never |  |  |  | Always |
| --- | --- | --- | --- | --- | --- | --- |
| C-1 Symptom management | 16.Correctly respond to emergency situations such as stroke occurrence / recurrence | 1 | 2 | 3 | 4 | 5 |
|  | 17. Correctly handle stroke sequelae / complications | 1 | 2 | 3 | 4 | 5 |
|  | 18. Inform the medical staff as soon as possible when medication side effects occur | 1 | 2 | 3 | 4 | 5 |
| C-2 Activities and rehabilitation management | 19. Do daily activities (e.g., washing, housework) within one's ability, as permitted by physical condition | 1 | 2 | 3 | 4 | 5 |
|  | 20. Participate in social life (e.g., participation in parties, return to work), as permitted by physical condition | 1 | 2 | 3 | 4 | 5 |
|  | 21. Adjust the way and intensity of exercise flexibly according to the condition, rehabilitation effect and environmental conditions | 1 | 2 | 3 | 4 | 5 |
| C-3 Emotional management | 22. Develop a positive attitude towards life ( e.g., avoid the idea that you are a burden, keep smiling) | 1 | 2 | 3 | 4 | 5 |
|  | 23. Do something to relieve stress and negative emotions (e.g., find someone to talk to, listen to music) | 1 | 2 | 3 | 4 | 5 |
