# Supplementary material for: The caregiver contribution to self-care of stroke inventory (CC-SCSI): evaluation of psychometric characteristics
Source: BMC Nurs. 2024 Apr 26;23:284. doi: 10.1186/s12912-024-01964-3 (PMC11055333; doi:10.1186/s12912-024-01964-3)
Supplement: Supplementary file 3 — Supplementary Material 3. [file 12912_2024_1964_MOESM3_ESM.docx]

**Supplementary file 2**

The Caregiver Contribution to Self-Care of Stroke Inventory (CC-SCSI) is designed based on the Self-Care of Stroke Inventory (SCSI) ^[1]^ to make it applicable for measurement within the caregiver population.

[1] Wang W, Mei Y, Vellone E, Zhang Z, Liu B, Zhou C, et al. Development and psychometric testing of the self-care of stroke inventory. Disabil Rehabil. 2023:1-10

**The Caregiver Contribution to Self-Care of Stroke Inventory (CCSCSI)**

Instructions：Please think about your experiences last month while completing this survey.

**Part A (Caregiver's Contribution to Self-Care Maintenance):** The following are behaviors that stroke survivors may adopt to promote physical and mental health. Please indicate how often do you recommend the patient you care to do the following behaviors? Or how often do you help the patient you care when he/she cannot do it independently.

|  | Items | Never |  |  |  | Always |
| --- | --- | --- | --- | --- | --- | --- |
| A-1 Daily health behaviors | 1. Recommend /help the patient to maintain normal weight (height/weight 2= 18.5~24 kg/m²) | 1 | 2 | 3 | 4 | 5 |
|  | 2. Recommend /help the patient to maintain over 30 minutes mild or moderate physical activity 3 or more times a week | 1 | 2 | 3 | 4 | 5 |
|  | 3. Recommend /help the patient to maintain healthy eating habits (e.g., Salt intake < 6g/d, have more fruits and vegetables) | 1 | 2 | 3 | 4 | 5 |
|  | 4. Recommend /help the patient to maintain a regular life schedule (e.g., early to bed and early to rise, avoid excessive fatigue) | 1 | 2 | 3 | 4 | 5 |
| A-2 Knowledge gaining | 5. Recommend /help the patient to master stroke-related information (e.g., etiology, outcome, treatment and rehabilitation of stroke) | 1 | 2 | 3 | 4 | 5 |
|  | 6. Recommend /help the patient to obtain stroke-related information through multiple channels ( e.g., mobile terminals, books, consultants ) | 1 | 2 | 3 | 4 | 5 |
| A-3 Treatment compliance | 7. Recommend /help the patient to take medicines following the doctor advised | 1 | 2 | 3 | 4 | 5 |
|  | 8. Recommend /help the patient to do rehabilitation exercises following the doctor advised | 1 | 2 | 3 | 4 | 5 |
|  | 9. Recommend /help the patient to do regular check-ups following the doctor advised | 1 | 2 | 3 | 4 | 5 |

**Part B（Caregiver's Contribution to Self-Care Monitor）:** The following are behaviors that stroke survivors may adopt to monitor their condition. Please indicate how often do you recommend the patient you care to do the following behaviors? Or how often do you help the patient you care when he/she cannot do it independently.

|  | Items | Never |  |  |  | Always |
| --- | --- | --- | --- | --- | --- | --- |
| B-1 Self-care Monitor | 10. Recommend /help the patient to monitor the signs and symptoms of stroke occurrence / recurrence ( e.g., dizzy, eye and mouth distortion, limb weakness on one side ) | 1 | 2 | 3 | 4 | 5 |
|  | 11. Recommend /help the patient to monitor the occurrence and development of stroke sequelae/complications (e.g., hemiplegia, pneumonia, pressure sores) | 1 | 2 | 3 | 4 | 5 |
|  | 12. Recommend /help the patient to monitor symptoms during daily activities (e.g., fatigue, pain) | 1 | 2 | 3 | 4 | 5 |
|  | 13. Recommend /help the patient to monitor changes in biochemical indicators such as blood pressure and blood sugar | 1 | 2 | 3 | 4 | 5 |
|  | 14. Recommend /help the patient to monitor medication effects and side effects | 1 | 2 | 3 | 4 | 5 |
|  | 15. Recommend /help the patient to monitor the effect of rehabilitation exercise | 1 | 2 | 3 | 4 | 5 |

**Part C（Caregiver's Contribution to Self-Care Management）:** The following are behaviors that stroke survivors may adopt to control physical symptoms and emotional changes. Please indicate how often you would recommend/help the patient you care for to do the following behaviors when they cannot do it independently when they experience symptoms or emotional changes.

|  | Items | Never |  |  |  | Always |
| --- | --- | --- | --- | --- | --- | --- |
| C-1 Symptom management | 16. Help the patient to correctly respond to emergency situations such as stroke occurrence / recurrence | 1 | 2 | 3 | 4 | 5 |
|  | 17. Help the patient to correctly handle stroke sequelae / complications | 1 | 2 | 3 | 4 | 5 |
|  | 18. Help the patient to inform the medical staff as soon as possible when medication side effects occur | 1 | 2 | 3 | 4 | 5 |
| C-2 Activities and rehabilitation management | 19. Recommend /help the patient to do daily activities (e.g., washing, housework) within one's ability, as permitted by physical condition | 1 | 2 | 3 | 4 | 5 |
|  | 20. Recommend /help the patient to participate in social life (e.g., participation in parties, return to work), as permitted by physical condition | 1 | 2 | 3 | 4 | 5 |
|  | 21. Recommend /help the patient to adjust the way and intensity of exercise flexibly according to the condition, rehabilitation effect and environmental conditions | 1 | 2 | 3 | 4 | 5 |
| C-3 Emotional management | 22. Recommend /help the patient to develop a positive attitude towards life ( e.g., avoid the idea that you are a burden, keep smiling) | 1 | 2 | 3 | 4 | 5 |
|  | 23. Recommend /help the patient to do something to relieve stress and negative emotions (e.g., find someone to talk to, listen to music) | 1 | 2 | 3 | 4 | 5 |
