# Supplementary material for: The caregiver contribution to self-care of stroke inventory (CC-SCSI): evaluation of psychometric characteristics
Source: BMC Nurs. 2024 Apr 26;23:284. doi: 10.1186/s12912-024-01964-3 (PMC11055333; doi:10.1186/s12912-024-01964-3)
Supplement: Supplementary file 4 — Supplementary Material 4. [file 12912_2024_1964_MOESM4_ESM.docx]

**Supplementary file 3**

**Interview guide**

| 1. Could you explain the specific challenges you faced in understanding certain parts of the questionnaire? |
| --- |
| 2. How could clearer definitions or examples have helped improve your understanding? |
| 3. Do you have any suggestions regarding the questions in this questionnaire or the explanations provided by the interviewer? How could they be improved to better assist you? |
| 4. After completing this questionnaire, what were your feelings, and do you think it will be helpful for your future care of patients as a caregiver? |
| 5. Do you have anything else you'd like to add or share regarding your experience? Your thoughts and feelings are important to us. |
